# Modulation of Higher‐order Behaviour in Model Protocell Communities by Artificial Phagocytosis

**DOI:** 10.1002/anie.201901469

**Published:** 2019-04-02

**Authors:** Laura Rodríguez‐Arco, B. V. V. S. Pavan Kumar, Mei Li, Avinash J. Patil, Stephen Mann

**Affiliations:** ^1^ Centre for Protolife Research and Centre for Organized Matter Chemistry School of Chemistry University of Bristol Bristol BS8 1TS UK

**Keywords:** colloids, emulsions, enzymes, microcapsules, protocells

## Abstract

Collective behaviour in mixed populations of synthetic protocells is an unexplored area of bottom‐up synthetic biology. The dynamics of a model protocell community is exploited to modulate the function and higher‐order behaviour of mixed populations of bioinorganic protocells in response to a process of artificial phagocytosis. Enzyme‐loaded silica colloidosomes are spontaneously engulfed by magnetic Pickering emulsion (MPE) droplets containing complementary enzyme substrates to initiate a range of processes within the host/guest protocells. Specifically, catalase, lipase, or alkaline phosphatase‐filled colloidosomes are used to trigger phagocytosis‐induced buoyancy, membrane reconstruction, or hydrogelation, respectively, within the MPE droplets. The results highlight the potential for exploiting surface‐contact interactions between different membrane‐bounded droplets to transfer and co‐locate discrete chemical packages (artificial organelles) in communities of synthetic protocells.

Artificial aqueous microcompartments capable of mimicking biological functions, such as encapsulation, selective exchange of chemicals with the environment, and minimal metabolism, are currently under investigation as model protocells in synthetic biology, origin‐of‐life studies, and biotechnology.^1^, ^2^, ^3^, ^4^, ^5^ The external membrane of these synthetic microcapsules can be tailored using a wide range of building blocks, such as lipids, polymers, protein–polymer conjugates, and inorganic nanoparticles, to meet specific criteria.^2^ For example, a protocell model based on the spontaneous assembly of partially hydrophobic silica nanoparticles at the interface of water and oil to form water‐in‐oil Pickering emulsion droplets with a mechanically robust membrane has been recently developed. The inorganic membrane was crosslinked to produce shell‐like micro‐compartments (colloidosomes) that could be transferred to water and endowed with biomimetic functions, such as in situ gene expression,^6^ enzyme‐mediated catalysis,^6^, ^7^ microcapsule growth and division,^8^ membrane gating,^9^ enzyme‐directed secretion of an extracellular‐like matrix,^10^ and energy capture and conversion.^11^

The emergence of collective behaviour in mixed populations of synthetic protocells is relatively unexplored, even though increasing levels of protocell interactivity could provide more complex and synergistic functions, such as resilience to environmental stress, chemical signalling for artificial quorum sensing, and multiplex tasking via collaboration and specialization. Chemical communication and signalling have been demonstrated between populations of vesicles and bacteria,^12^, ^13^ lipid vesicles containing gene circuitry,^14^ vesicles and proteinosomes,^15^ colloidosomes,^16^ modified^17^ or immobilized^18^ coacervate droplets, and in water‐in‐oil emulsion droplets.^19^, ^20^, ^21^ Hierarchically structured compartments involving different types of protocells, such as liposome‐encapsulated coacervate droplets,^22^ multi‐compartmentalized polymersomes,^23^ nested vesicles,^24^ and giant vesicles comprising light‐harvesting organelles,^25^ have also been reported. Contact‐induced adhesive interactions between proteinosomes^26^ or lipid‐coated emulsion droplets^27^ have been developed to produce aggregates of protocells with coordinated functions (prototissues), and sophisticated population dynamics used as mechanisms of artificial predation^28^ and parasitism^29^ in dispersed binary protocell populations.

We recently developed a primitive form of artificial phagocytosis in a size‐mismatched binary population of water‐in‐oil Pickering emulsion droplets in which cross‐linked semi‐permeable silica nanoparticle‐stabilized colloidosomes were spontaneously ingested by larger magnetic iron oxide Pickering emulsion (MPE) droplets comprising particle‐free fatty acid stabilized apertures.^30^ The particle‐free patches were formed by addition of oleic acid to the oil (dodecane) phase (typically 2 mg mL^−1^), which partially destabilized the iron oxide shell by changing the oil–water interfacial tension at the surface of the MPE droplets. Engulfment of the silica colloidosomes through the apertures was associated with the formation of a surface‐adsorbed oleate bilayer, which was facilitated by maintaining a pH within the MPE droplets close to the fatty acid p*K*_a_ (9.8).^30^ Significantly, small water‐soluble molecules initially entrapped within the colloidosomes were released into the MPE droplets after phagocytosis. As a consequence, by initially encapsulating an enzyme substrate and complementary enzyme in the colloidosomes and MPE droplets, respectively, a dephosphorylation reaction could be triggered in the aqueous phase of the magnetic microcapsules. Herein, we extend this approach to the delivery of enzymes contained in the silica colloidosomes as a way to induce higher‐order structural and functional changes inside the MPE droplet host via artificial phagocytosis. As the macromolecular payloads are too large to diffuse through the pores of the silica nanoparticle membrane, the enzymes are delivered in the form of discrete chemical packages (artificial “organelles”) with minimal contamination of the internal solution of the MPE droplets. Specifically, we prepare catalase‐filled colloidosomes capable of activating the buoyant motion of hydrogen peroxide containing MPE droplets, lipase‐entrapped colloidosomes that trigger their own phagocytosis by changing the interfacial tension of the MPE droplets via in situ triglyceride hydrolysis, and alkaline phosphatase‐containing colloidosomes that initiate the nucleation and growth of a supramolecular hydrogel network inside amino acid‐containing MPE droplets. These higher‐level changes are relevant in the context of protocell community dynamics, enabling phenomena such as the spontaneous segregation of two populations, self‐triggering of phagocytosis/viral‐like behaviour, and arrestment of phagocytosis by a prey population. Taken together, our results show that the dynamics of model protocell communities can be exploited to modulate the function and higher‐order behaviour of mixed populations of micro‐compartmentalized colloidal objects in response to internal triggers.

Phagocytosis‐inspired behaviour was induced by addition of oleic acid to a dodecane dispersion of MPE droplets (mean size=500±250 μm; pH 10.2) and silica colloidosomes (mean size=52±10 μm; pore size ca. 3–4 nm; Supporting Information, Figure S1).^30^ By encapsulating catalase and hydrogen peroxide (H_2_O_2_) in the silica colloidosomes and MPE droplets, respectively, ingestion of the colloidosomes (Supporting Information, Figure S2) triggered the almost instantaneous enzyme‐mediated production of micrometre‐sized oxygen bubbles inside the MPE droplets (Figure 1 a–c; Supporting Information, Movie S1) provided that the encapsulated H_2_O_2_ and catalase concentrations were higher than 1 % and 1 mg mL^−1^, respectively (Figure 1 d). Bubble generation in MPE droplets containing 1 % H_2_O_2_ increased from ca. 20 to 50 % when the entrapped catalase concentration was increased from 5 to 30 mg mL^−1^ whilst at 5 % H_2_O_2_ essentially all the MPE droplets contained gas bubbles (Supporting Information, Figure S3a). Although the oxygen bubbles were exclusively generated inside the aqueous interior of the MPE droplets, they were often released to the external environment through the fatty‐acid stabilized patches in the magnetic membrane (Supporting Information, Movie S2). Nevertheless, for concentrations of H_2_O_2_ higher than 2 % (Supporting Information, Figure S3b), nucleation and formation of bubbles occurred faster than their release to the external oil phase, leading to accumulation within the MPE droplets and buoyancy (Figure 1 e; Supporting Information, Figure S3b, Movie S3). Significantly, the formation of gas bubbles and generation of buoyancy required an interaction between the two protocell populations via phagocytosis in contrast to previous results involving the buoyant motion of a single population of organoclay/DNA protocells.^31^ The buoyant translational motion was characterized by well‐defined vertical trajectories towards the oil/air interface with an average speed of around 1.5 cm s^−1^. Phagocytosis of one single colloidosome was often enough to generate buoyant MPE droplets for levels of encapsulated catalase above 30 mg mL^−1^, while several capture events were required to float the MPE droplets at lower enzyme concentrations (Figure 1 f). As a consequence, depending on the entrapped enzyme concentration, deposition of the MPE droplets onto a highly crowded field of sedimented colloidosomes resulted in almost instantaneous or delayed (ca. 20 s at 1 mg mL^−1^) buoyancy‐induced segregation of the two protocell populations (Supporting Information, Figure S4).

**Figure 1:**
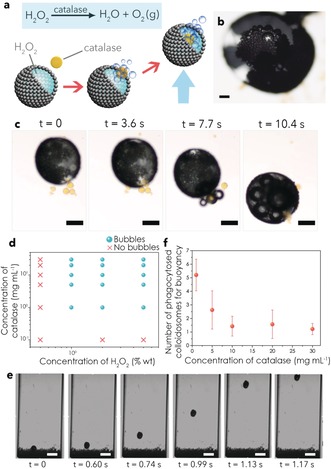
a) Generation of buoyant gas bubbles inside H_2_O_2_‐containing MPE droplets via phagocytosis of catalase‐containing silica colloidosomes. Accumulation of oxygen bubbles within the MPE droplets results in buoyancy (indicated by blue arrow). b) Optical microscopy image showing oxygen bubbles inside an MPE droplet (2 % H_2_O_2_) after phagocytosis of catalase‐containing colloidosomes; scale bar=100 μm. c) Time sequence of optical microscopy images showing spontaneous engulfment of several catalase‐containing silica colloidosomes (yellow objects, 30 mg mL^−1^) by a MPE droplet containing a 5 % solution of H_2_O_2_ to produce oxygen bubbles (small dark objects) and buoyancy of the MPE droplet. See the Supporting Information, Movie S1 for complete sequence; scale bar=200 μm; yellow colouration is due to co‐encapsulation of the dye calcein in the colloidosomes. d) Phase plot indicating the presence (blue spheres) or absence (red crosses) of oxygen bubbles in phagocytosis experiments undertaken at different concentrations of H_2_O_2_ inside the MPE droplets (X‐axis), and of catalase inside the silica colloidosomes (Y‐axis). e) Time sequence of optical microscopy images showing buoyancy and vertical displacement of a MPE droplet with 5 % H_2_O_2_ after phagocytosis of silica colloidosomes containing 30 mg mL^−1^ catalase. See the Supporting Information, Movie S3 for complete sequence; scale bar=1 mm. f) Plot showing the number of phagocytosed catalase‐entrapping colloidosomes required to induce buoyant motion of MPE droplets (5 % H_2_O_2_) vs. concentration of catalase. Error bars are based on binomial proportion confidence intervals for nine different droplets. White balance correction has been applied to (b), (c), and (e).

We sought to increase the complexity of the above artificial phagocytosis process by developing a strategy that would enable in situ triggering of protocell ingestion by auxiliary enzyme activity in the guest colloidosomes rather than relying on the external addition of oleic acid. For this, we prepared lipase‐containing silica colloidosomes, added them to a population of MPEs dispersed in a dodecane solution of a triglyceride (triolein), and investigated whether enzyme‐mediated hydrolysis of triolein within the colloidosomes and concomitant release of surface‐active agents (mono/diacyl glycerols, oleic acid) could induce the opening of apertures in the shell of initially intact MPE droplets (Figure 2 a). Control experiments showed that the shell of the MPE droplets remained immobilized and closed when dispersed in a 20 mg mL^−1^ triolein solution in the presence or absence of lipase‐free colloidosomes (Supporting Information, Figure S5). In contrast, when lipase‐containing silica colloidosomes were added to the dodecane phase, the fluidity of the iron oxide membrane progressively increased such that particle‐free domains were produced within the MPE shell (Figure 2 b; Supporting Information, Movie S4). Formation of the particle‐free apertures was dependent on the triolein and lipase concentrations (Figure 2 c; Supporting Information, Figure S6a), consistent with a subsequent decrease of the oil–water interfacial tension at the surface of the MPE droplets owing to the production of mono/diacylglycerols and oleic acid (Figure 2 d; Supporting Information, Figures S6b and S7). Significantly, segregation of the iron oxide membrane resulted in slow penetration of the lipase‐containing colloidosomes into the aqueous phase of the MPE droplets through the particle‐free domains exposed at the water‐droplet–oil interface (Figure 2 b; Supporting Information, Movie S4). Transfer into the host interior occurred more slowly compared with when the colloidosomes were initially dispersed in a 2 mg mL^−1^ solution of oleic acid in dodecane (ca. 2 min vs. 3 s, respectively) even though the decrease in oil/water interfacial tension with surfactant concentration was similar for oleic acid and a monoacylglycerol such as monoolein (1‐oleoyl‐*rac*‐glycerol; Supporting Information, Figure S8).^32^ Control experiments indicated that both surfactants produced particle‐free apertures when added individually to dodecane dispersions of the MPE droplets, but that the rates of colloidosome penetration were negligible for monoolein. As transfer into the water phase of the MPE droplets is dependent on the formation of a surfactant bilayer/multilayer at the surface of the colloidosomes,^30^ we attributed the difference in phagocytosis efficiency to the facile interfacial assembly of oleic acid compared with mono‐ and diacylglycerols.

**Figure 2:**
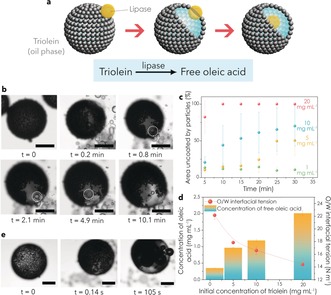
a) The use of lipase‐containing colloidosomes for triggering artificial phagocytosis. Lipase‐mediated hydrolysis of triolein releases surface‐active molecules that produce particle‐free apertures in the MPE droplet membrane followed by engulfment of the colloidosomes. b) Time sequence of optical microscopy images showing the formation of a particle‐free aperture in the initially intact shell of a MPE droplet (pH 10.2) dispersed in a dodecane solution of triolein (10 mg mL^−1^) after addition of multiple silica colloidosomes containing encapsulated lipase (100 U mL^−1^). The silica colloidosomes in contact with the particle‐free patches of the magnetic droplet are spontaneously transferred into the aqueous phase. See the Supporting Information, Movie S4 for complete sequence. Scale bar=100 μm. c) Plot showing time‐dependent percentage changes in particle‐free surface area for MPE droplets against triolein concentration after addition of silica colloidosomes containing encapsulated lipase (100 U mL^−1^). Increased levels of triolein hydrolysis are associated with larger apertures in the MPE droplets. d) Plots of the concentration of free oleic acid (column plot, left Y axis) and oil/water interfacial tension (scatter plot, right Y axis) against the initial concentration of triolein in dodecane after the addition of lipase‐containing colloidosomes (10 000 U mL^−1^). The concentration of released oleic acid increases with the initial substrate concentration resulting in a decrease of the interfacial tension responsible for the formation of particle‐free domains in the MPE droplet membrane. Error bars correspond to standard deviations. e) Time sequence of optical microscopy images showing the formation of a membrane aperture and bubble formation in an initially intact MPE droplet (pH 10.2) after addition of multiple silica colloidosomes containing both lipase (10 kU mL^−1^) and catalase (30 mg mL^−1^). Scale bar=150 μm. White balance correction and increase of brightness have been applied to (b) and (e).

Given the above observations, we co‐encapsulated catalase and lipase within the silica colloidosomes to produce a hybrid protocell capable of triggering phagocytosis and inducing buoyancy (or disintegration) of the host MPE droplet. Optical microscope images showed that upon addition of the enzyme‐containing silica colloidosomes to a suspension of 5 % H_2_O_2_‐containing MPE droplets (carbonate buffer, 1 m, pH 10.2) dispersed in a 40 mg mL^−1^ dodecane solution of triolein, the magnetic membrane was rapidly opened and bubbles were progressively formed in the aqueous phase lumen to produce buoyant MPE droplets (Figure 2 e; Supporting Information, Movie S5).

Finally, we used the process of artificial phagocytosis to trigger in situ structural reconfiguration of the MPE/colloidosome host–guest microcompartments. For this, we mixed a population of alkaline phosphatase (ALP)‐containing silica colloidosomes with a dispersion of MPE droplets prepared from an aqueous solution of the phosphorylated amino acid precursor, *N*‐fluorenylmethyloxycarbonyl‐tyrosine‐(*O*)‐phosphate (Fmoc‐TyrP) as a means of generating the phagocytosis‐mediated self‐assembly of a supramolecular hydrogel network within the confined interior of the protocell construct. Engulfment of the silica colloidosomes resulted in ALP‐mediated dephosphorylation of Fmoc‐TyrP and self‐assembly of *N*‐fluorenylmethyloxycarbonyl‐tyrosine (Fmoc‐TyrOH) into a filamentous hydrogel (Figure 3 a). Optical and fluorescence microscopy images recorded in the presence of the hydrogel binding dye Hoechst 33 258 showed blue fluorescence associated with the engulfed colloidosomes (Figure 3 b–e). The fluorescence intensity increased rapidly within 1 h of engulfment, after which there was a slow increase over 24 h (Figure 3 f). Ten‐fold changes in the concentration of either the substrate or enzyme had minimal effect on the final outcome of the hydrogelation reaction after 24 h (Supporting Information, Figure S9). We attributed this to the spatial restrictions placed on hydrogel growth owing to confinement within the limited aqueous volumes of the MPE/colloidosome host–guest microcompartments. Hoechst 33258 fluorescence was mainly associated with the colloidosome interior (Figure 3 d), consistent with diffusion of the FmocTyrP molecules through the cross‐linked silica nanoparticle membrane and hydrogelation in close proximity to the entrapped ALP. Blue fluorescence was also observed in the associated MPE droplet phase and in partially engulfed or surface‐connected silica colloidosomes (Supporting Information, Figure S10), indicating a counter‐flow of Fmoc‐TyrOH monomers from the colloidosomes into the surrounding aqueous microenvironments. When left to dry overnight, the hydrogelled MPE droplets remained intact, while in the absence of phagocytosis the MPE droplets irreversibly collapsed (Supporting Information, Figure S11). Scanning electron microscopy (SEM) images of dried MPE droplets recorded 7 days after the onset of hydrogelation showed spheroidal porous microcompartments (Figure 3 g) with a shell comprising a network of iron oxide particles, hydrogel filaments, and embedded silica colloidosomes (Figure 3 g; Supporting Information, Figure S12). Immobilization of the iron oxide particles in the hydrogel prevented any further membrane restructuring of the MPE droplets by addition of a dodecane solution of oleic acid (15 mg mL^−1^).

**Figure 3:**
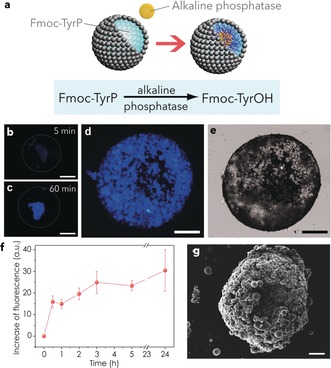
a) The phagocytosis‐induced internal hydrogelation of Fmoc‐TyrP‐containing MPE droplets. Engulfment of ALP‐containing colloidosomes leads to dephosphorylation of Fmoc‐TyrP and self‐assembly of a supramolecular hydrogel. b)–d) Fluorescence microscopy images showing single Fmoc‐TyrP (10 mm; pH 10.2)‐containing MPE droplets 5 min (b), 1 h (c) and 24 h after phagocytosis of multiple ALP‐containing silica colloidosomes (31 U mL^−1^). The blue fluorescence is associated with binding of Hoechst 33258 to the supramolecular hydrogel assembled within the ingested colloidosomes; scale bar=200 μm. e) Corresponding bright‐field microscopy image of sample displayed in (d) showing high density of intact colloidosomes within the single MPE droplet; scale bar=200 μm. (f) Plot showing time‐dependent increase in blue fluorescence accompanying phagocytosis of ALP‐containing colloidosomes into FmocTyr‐P‐containing MPE droplets ([Fmoc‐TyrP]=2 mm; [ALP]=31 U mL^−1^) and concomitant hydrogelation. Bars on data points represent standard deviations. g) Scanning electron micrograph of a dried single Fmoc‐TyrP‐containing MPE droplet after phagocytosis of multiple ALP‐encapsulating colloidosomes (intact small spheres) showing a hydrogelled network of iron oxide particles and embedded silica colloidosomes. Scale bar=100 μm. Increase of brightness has been applied to (g).

In conclusion, we have demonstrated that higher‐order structural and functional changes can be enzymatically activated in a binary population of bioinorganic protocells consisting of MPE droplets engineered to artificially phagocytose enzyme‐containing silica colloidosomes. Phagocytosis of catalase‐containing colloidosomes catalyses the decomposition of hydrogen peroxide encapsulated inside the MPE droplets to produce oxygen bubbles that give rise to buoyancy‐induced vertical motion and removal of the MPE droplet population from the mixed protocell community. Alternatively, encapsulation of lipase in the silica colloidosomes triggers the phagocytosis response by generating particle‐free domains in the MPE droplet membrane via colloidosome‐mediated hydrolysis of triolein to produce free oleic acid in the continuous oil phase. Combining these two strategies, encapsulation of both catalase and lipase inside the silica microcontainers transforms them into “viral” protocells capable of self‐triggered phagocytosis and removal of the MPE proto‐phagocyte population. Finally, we have demonstrated that the artificial phagocytosis of silica colloidosomes containing encapsulated ALP generates an internal hydrogel network by enzyme‐mediated dephosphorylation of an amino acid gel precursor trapped inside the MPE droplets. Taken together our results highlight the potential of exploiting surface‐contact interactions between different membrane‐bounded droplets to trigger encapsulated biochemical reactions that give rise to higher‐order behaviours in protocell consortia. Although our observations are exploratory, collective interactivity between compartmentalized microscale objects could provide a step towards new applications in biomimetic storage and delivery, and microreactor technology.

## Conflict of interest

The authors declare no conflict of interest.

## Supporting information

As a service to our authors and readers, this journal provides supporting information supplied by the authors. Such materials are peer reviewed and may be re‐organized for online delivery, but are not copy‐edited or typeset. Technical support issues arising from supporting information (other than missing files) should be addressed to the authors.

Supplementary

Supplementary

Supplementary

Supplementary

Supplementary

Supplementary

## References

[1] Y. Tu , F. Peng , A. Adawy , Y. Men , L. K. E. A. Abdelmohsen , D. A. Wilson , Chem. Rev. 2016, 116, 2023. 26583535 10.1021/acs.chemrev.5b00344

[2] M. Li , X. Huang , T.-Y. D. Tang , S. Mann , Curr. Opin. Chem. Biol. 2014, 22C, 1. 10.1016/j.cbpa.2014.05.018 24952153

[3] L. Schoonen , J. C. M. van Hest , Adv. Mater. 2016, 28, 1109. 26509964 10.1002/adma.201502389

[4] B. T. Kelly , J. C. Baret , V. Taly , A. D. Griffiths , Chem. Commun. 2007, 1773. 10.1039/b616252e 17476389

[5] A. Küchler , M. Yoshimoto , S. Luginbühl , F. Mavelli , P. Walde , Nat. Nanotechnol. 2016, 11, 409. 27146955 10.1038/nnano.2016.54

[6] M. Li , D. C. Green , J. L. R. Anderson , B. P. Binks , S. Mann , Chem. Sci. 2011, 2, 1739.

[7] C. Wu , S. Bai , M. B. Ansorge-Schumacher , D. Wang , Adv. Mater. 2011, 23, 5694. 22072496 10.1002/adma.201102693

[8] M. Li , X. Huang , S. Mann , Small 2014, 10, 3291. 24861579 10.1002/smll.201400639

[9] M. Li , R. L. Harbron , J. V. Weaver , B. P. Binks , S. Mann , Nat. Chem. 2013, 5, 529. 23695636 10.1038/nchem.1644

[10] K. Akkarachaneeyakorn , M. Li , S. A. Davis , S. Mann , Langmuir 2016, 32, 2912. 26981922 10.1021/acs.langmuir.6b00553

[11a] S. Wang , M. Li , A. J. Patil , S. Sun , L. Tian , D. Zhang , M. Cao , S. Mann , J. Mater. Chem. A 2017, 5, 24612.

[12] P. M. Gardner , K. Winzer , B. G. Davis , Nat. Chem. 2009, 1, 377. 21378891 10.1038/nchem.296

[13] R. Lentini , et al., Nat. Commun. 2014, 5, 4012. 24874202 10.1038/ncomms5012 PMC4050265

[14] K. P. Adamala , D. A. Martin-Alarcon , K. R. Guthrie-Honea , E. S. Boyden , Nat. Chem. 2017, 9, 431. 28430194 10.1038/nchem.2644 PMC5407321

[15] T-Y. D. Tang , D. Cecchi , G. Fracasso , D. Accardi , A. Coutable-Pennarun , S. S. Mansy , A. W. Perriman , J. L. R. Anderson , S. Mann , ACS Synth. Biol. 2018, 7, 339. 29091420 10.1021/acssynbio.7b00306

[16] S. Sun , M. Li , F. Dong , S. Wang , L. Tian , S. Mann , Small 2016, 12, 1920. 26923794 10.1002/smll.201600243

[17] D. S. Williams , A. J. Patil , S. Mann , Small 2014, 10, 1830.24515342

[18] L. Tian , M. Li , J. Liu , A. J. Patil , B. W. Drinkwater , S. Mann , ACS Cent. Sci. 2018, 4, 1551. 30555908 10.1021/acscentsci.8b00555 PMC6276052

[19] M. Weitz , J. Kim , K. Kapsner , E. Winfree , E. Franco , F. C. Simmel , Nat. Chem. 2014, 6, 295. 24651195 10.1038/nchem.1869

[20] M. Schwarz-Schilling , L. Aufinger , A. Muckl , F. C. Simmel , Integr. Biol. 2016, 8, 564. 10.1039/c5ib00301f 26778746

[21] A. Dupin , F. C. Simmel , Nat. Chem. 2019, 11, 32. 30478365 10.1038/s41557-018-0174-9 PMC6298583

[22] N. N. Deng , W. T. S. Huck , Angew. Chem. Int. Ed. 2017, 56, 9736; 10.1002/anie.201703145 PMC5601218 28658517

[23] R. J. R. W. Peters , M. Marguet , S. Marais , M. W. Fraaije , J. C. M. van Hest , S. Lecommandoux , Angew. Chem. Int. Ed. 2014, 53, 146; 10.1002/anie.201308141 24254810

[24] J. W. Hindley , Y. Elani , C. M. McGilvery , S. Ali , C. L. Bevan , R. V. Law , O. Ces , Nat. Commun. 2018, 9, 1093. 29545566 10.1038/s41467-018-03491-7 PMC5854585

[25] K. Y. Lee , L. Mahadevan , S.-J. Park , K. A. Lee , S.-H. Kim , H. Kim , Y. Meroz , K.-H. Jung , T. K. Ahn , K. K. Parker , K. Shin , Nat. Biotechnol. 2018, 36, 530. 29806849 10.1038/nbt.4140

[26] P. Gobbo , A. J. Patil , M. Li , S. Mann , Nat. Mater. 2018, 17, 1145. 30297813 10.1038/s41563-018-0183-5

[27] M. J. Booth , V. R. Schild , A. D. Graham , S. N. Olof , H. Bayley , Sci. Adv. 2016, 2, e1600056. 10.1126/sciadv.1600056 PMC4820383 27051884

[28] Y. Qiao , M. Li , R. Booth , S. Mann , Nat. Chem. 2016, 9, 110. 28282044 10.1038/nchem.2617

[29] N. Martin , J.-P. Douliez , Y. Qiao , R. Booth , M. Li , S. Mann , Nat. Commun. 2018, 9, 3652. 30194369 10.1038/s41467-018-06087-3 PMC6128866

[30] L. Rodríguez-Arco , M. Li , S. Mann , Nat. Mater. 2017, 16, 857. 28604713 10.1038/nmat4916

[31] B. V. V. S. P. Kumar , A. J. Patil , S. Mann , Nat. Chem. 2018, 10, 1154. 30127511 10.1038/s41557-018-0119-3

[32] K. Kleberg , F. Jacobsen , D. G. Fatouros , A. J. Müllertz , Pharm. Sci. 2010, 99, 3522. 10.1002/jps.22122 20564382

