# Supplementary material for: Modulation of Higher‐order Behaviour in Model Protocell Communities by Artificial Phagocytosis
Source: Angew Chem Int Ed Engl. 2019 Apr 2;58(19):6333–7. doi: 10.1002/anie.201901469 (PMC6519160; doi:10.1002/anie.201901469)
Supplement: Supplementary file 1 — Supplementary [file ANIE-58-6333-s001.pdf]

Angewandte Chemie
Eine Zeitschrift der Gesellschaft Deutscher Chemiker

Supporting Information

Modulation of Higher-order Behaviour in Model Protocell Communities by Artificial Phagocytosis

*Laura Rodríguez-Arco, B. V. V. S. Pavan Kumar, Mei Li, Avinash J. Patil, and Stephen Mann**

anie_201901469_sm_miscellaneous_information.pdf
anie_201901469_sm_Movie_S1.mp4
anie_201901469_sm_Movie_S2.mp4
anie_201901469_sm_Movie_S3.mp4
anie_201901469_sm_Movie_S4.mp4
anie_201901469_sm_Movie_S5.mp4

Supporting Information: Methods, Movies and Supplementary Figures

1. Methods

Preparation of magnetic Pickering emulsion droplets. Water-in-oil magnetic Pickering emulsion (MPE) droplets were prepared as described previously (Rodríguez-Arco, L., Li, M. & Mann, S. *Nat. Mater.* **16**, 857-863 (2017)). Briefly, 5 mg of partially hydrophobic oleate-capped magnetite particles were dispersed in 1 mL of dodecane (purity ≥ 99%, Sigma-Aldrich, UK) followed by sonication for 1 min prior to the addition of 50 µL of the aqueous phase (0.1 M carbonate buffer, pH 10.2) in an Eppendorf tube. The mixture was then stirred for 15 s at 25,000 rpm using an UltraTurrax T 10 homogenizer with a S10 D-7G-KS-65 dispersing tip (IKA, Germany). A range of solutes (see below) were encapsulated within the droplets to study the activation of structural/functional changes driven by the phagocytosis of silica colloidosomes.

Preparation of silica colloidosomes. Silica colloidosomes were formed according to a previously reported procedure (Li, M., et al. *Chem. Sci.* **2**, 1739-1745 (2011)). For the preparation, 15 mg of hydrophobic silica nanoparticles (Wacker-Chemie, Germany) were dispersed in 2 mL of dodecane and vortexed for 30 s. The aqueous phase (100 µL) was then added and the mixture was stirred with an UltraTurrax T18 homogenizer (IKA, Germany) for 30 s at 10,000 rpm. Several enzymes were encapsulated within the silica colloidosomes to investigate the activation of structural/functional changes within the MPE droplets (see below). In order to protect the colloidosomes from disassembly upon phagocytosis, the silica membrane was crosslinked by the addition of 25 µL of tetramethoxysilane (TMOS, 98%, Sigma-Aldrich, USA). After this step, the colloidosomes were stirred overnight in a rotator at 4 °C and kept at this temperature for storage before use.

Enzyme labelling. For fluorescent labelling, 5 mg of the enzymes were dissolved in 2 mL of carbonate buffer (0.1 M, pH 8.5) followed by the dropwise addition of 50 µL of either fluorescein isothiocyanate or rhodamine B isothiocyanate (Sigma-Aldrich, UK) solutions in DMSO (1 mg mL^{-1}). The mixture was then stirred at room temperature for 5 h, dialyzed against MilliQ water for 48 h with frequent changes of water, and freeze dried.

Phagocytosis-triggered bubble generation within magnetic Pickering emulsion droplets and buoyancy. The enzyme-mediated decomposition of hydrogen peroxide (H_2O_2) within the MPE droplets to generate oxygen bubbles was achieved by encapsulating a solution (5-30 mg mL^{-1} in 0.1 M carbonate buffer, pH 10.2) of catalase (250 kDa; from bovine liver, ≥ 10,000 U mg^{-1}, Sigma-Aldrich, UK) within the cross-linked silica colloidosomes. A 5 mg mL^{-1} solution of the dye calcein (Sigma-Aldrich, UK) was co-encapsulated with catalase to give the colloidosomes a yellow colour. In a typical experiment, a dodecane suspension of the colloidosomes (200 µL, water/dodecane volume ratio = 0.05, silica/water weight ratio = 0.15, 25 µL TMOS) was pipetted and placed in a polystyrene Petri dish (3.5 cm diameter, Corning, USA) containing 1 mL of a 2 mg mL^{-1} solution of oleic acid in dodecane. Then, 20 µL of a water-in-dodecane magnetite Pickering emulsion (water/dodecane = 0.05, magnetite/water weight ratio = 0.1) entrapping a solution (0.5-5 wt %) of H_2O_2 (Fisher-Scientific, UK) in carbonate buffer (pH 10.2) were added, and the sample was investigated using an Olympus BX53 optical microscope (Olympus, Japan). The behaviour of nine different droplets was monitored immediately after for each concentration pair of catalase-H_2O_2. The probability of bubble formation was estimated by quantifying the number of droplets within which bubbles were detected and dividing this value by the total number of droplets imaged in the same experimental conditions. Similar estimations were made for the probability of buoyancy occurrence. The corresponding error bars in these probability experiments were based on binomial proportion confidence intervals. In addition,

the number of phagocytosed colloidosomes required to generate buoyant motion of the MPE droplets was quantified by counting the number of colloidosomes incarcerated inside the MPE droplets before they initiated the vertical motion. Remote manipulation of the magnetic droplets to attract them to the colloidosomes was achieved by holding a neodymium cylindrical magnet (length = 6 mm, diameter = 5 mm; RS Components, UK) attached to a thin spatula (10 cm in length) in close proximity to them.

In a different experiment, 400 µL of the catalase-containing silica colloidosomes were carefully deposited at the bottom of a 4 mm-side glass square cuvette (height = 4.5 cm) filled with 4 mL of a 2 mg mL^{-1} oleic acid solution in dodecane, until complete coverage of the bottom with the colloidosomes. The cuvette was then held vertically with a clamp and a stand, and placed near the 1.25 x objective of an Axioskop optical microscope (Carl Zeiss, USA) which was tilted so that its main axis was perpendicular to the cuvette longest axis to allow for vertical imaging of the sample. Individual MPE droplets encapsulating 5 % solutions of H_2O_2 in carbonate buffer (water/dodecane = 0.05, magnetite/water weight ratio = 0.1, pH 10.2) were pipetted and dropped inside the cuvette. The time for the MPE droplets to start their buoyant motion since the moment they first hit the bottom of the cuvette was quantified by analysis of the optical microscope images. Nine MPE droplets were analysed for every concentration of catalase.

Lipase-triggered opening of magnetic Pickering emulsion droplets and phagocytosis. MPE droplets (water/dodecane = 0.05, magnetite/water weight ratio = 0.1, pH 10.2) were added to a polystyrene Petri dish filled with 1 mL of glyceryl trioleate (Sigma-Aldrich, purity ≥ 99 %) solutions in dodecane (1, 5, 10 and 20 mg mL^{-1}). After that, 25 µL of silica colloidosomes (water/dodecane volume ratio = 0.05, silica/water weight ratio = 0.15, 25 µL TMOS) entrapping 100 or 10,000 U mL^{-1} solutions of a lipase (49 kDa from *Aspergillus oryzae*, 50 U mg^{-1}, Sigma-Aldrich, UK) were pipetted and deposited on top of the MPE droplets. Changes in the percentage coverage of the magnetite particles in the membrane of MPE droplets were investigated by optical microscopy. Images of 5 different droplets were recorded at 5 min time intervals and were subsequently analyzed with the free software *ImageJ* to quantify the area of the particle-free domains in the magnetic membrane. Because the 2-D optical projections of the droplets in optical microscopy images do not allow for consideration of the droplet curvature, the data were plotted as the quotient of the *bare* droplet area (*i.e.*, free of particles) and the area of the whole droplet, in order to minimize the associated inaccuracies. The experiments were conducted in duplicate.

In a different experiment, lipase (200 mg mL^{-1}) and catalase (30 mg mL^{-1}) solutions in carbonate buffer (0.1 M, pH 10.2) were encapsulated together in the silica colloidosome population (water/dodecane = 0.05, magnetite/water weight ratio = 0.1) with the aim of studying the coupled effects of both enzymes, *i.e.*, self-induced phagocytosis and buoyancy. In this set of experiments, a single MPE droplet encapsulating a 5 % solution of H_2O_2 in carbonate buffer (1 M, pH 10.2) was deposited on a Petri dish filled with 1 mL of a 40 mg mL^{-1} triolein solution in dodecane. After this, 25 µL of the silica colloidosome dispersion were added and the enzyme-induced changes occurring in the magnetic droplets were studied by optical microscopy.

Interfacial tension measurements. The interfacial tension between monolein or oleic acid solutions of same molarity (3.5 to 530 µM) in dodecane and carbonate buffer was measured using the pendant drop method at 20 °C with a DSA100 tensiometer (Krüss, Germany). Briefly, drops of the aqueous phase were formed using a syringe and their change of shape with time was monitored for 1200 s. The interfacial tension was estimated using the Young-Laplace equation. The measurements were performed in duplicate.

The reduction of the interfacial tension between dodecane and carbonate buffer (0.1 M, pH 10.2) due to the hydrolysis of triolein by the lipase-encapsulated silica colloidosomes (lipase concentration 200

mg mL^{-1}) was also measured. For this, 75 μL of a colloidosome suspension in dodecane were added to 1.25 mL of a triolein solution (1-50 mg mL^{-1}) and the mixture was rotated for 1 h at room temperature. After that, the mixture was centrifuged at 3000 rcf for 5 min to separate the colloidosomes and to stop the hydrolysis, and the supernatant was collected. For the interfacial tension measurements, the supernatant was diluted in pure dodecane with a dilution ratio of 1:100. The experiments were carried out in duplicate.

Determination of the concentration of free oleic acid after lipase-mediated hydrolysis of triolein. The concentration of free oleic acid after the hydrolysis of triolein by the lipase-entrapped colloidosomes was quantified by the colorimetric assay (Kwon, D. Y. & Rhee, J. J. *Am. Oil Chem. Soc.* **63**, 89-92 (1986)). For this purpose, 75 μL of a colloidosome suspension in dodecane were added to 1.25 mL of a triolein solution (1-20 mg mL^{-1}) and the sample was rotated for 1 h at room temperature. After that, the hydrolysis was stopped by removing the lipase colloidosomes by centrifugation at 3000 rcf for 5 min, and 1 mL of the supernatant was added to an Eppendorf tube containing 200 μL of a copper reagent. The copper reagent consisted of a 5% solution of cupric acetate (Sigma-Aldrich, UK) at pH 6.1 (adjusted by the addition of pyridine, Sigma-Aldrich, UK). The mixture was vortexed for 90 s and left to stand still for 10 min to allow separation of the two phases. The absorbance at 715 nm of the dodecane phase was measured with a UV-Vis nanophotometer (Geneflow, IMPLEN, Denmark) and the concentration of free oleic acid was estimated from a calibration curve obtained using pure oleic acid-in-dodecane solutions.

Phagocytosis-triggered dephosphorylation and hydrogelation of the MPE aqueous core. Two hundred microliters of silica colloidosomes (water/dodecane volume ratio = 0.05, silica/water weight ratio = 0.15, 25 μL TMOS) filled with 31 or 310 mU mL^{-1} solutions of alkaline phosphatase, ALP (69 kDa, from calf intestine, CALBIOCHEM, Merck, Germany) were dispersed in 1 mL of a 2 mg mL^{-1} solution of oleic acid in dodecane and placed in a polystyrene Petri dish. After that, 20 μL of MPE droplets (water/dodecane = 0.05, magnetite/water weight ratio = 0.1, pH 10.2) encapsulating 2-20 mM solutions of the substrate fluorenymethylcarbonyl-tyrosine-(O)-phosphate (FMOC-TyrP, Novabiochem, carbonate buffer pH 10.2) were added, and the mixture was left at rest for 30 min. The dephosphorylation of FMOC-Tyr-P to fluorenymethylcarbonyl-tyrosine (FMOC-TyrOH) and gelation of the MPE droplet lumen was followed by monitoring the time-dependent increase of blue fluorescence associated to binding of Hoechst 33258 dye (1 mM, emission wavelength = 461 nm, Sigma-Aldrich, UK) to the FMOC-TyrOH nanofilaments using a Leica DMI3000 B microscope (Leica, Germany). Five different droplets were imaged at each time point for all the substrate and enzyme concentrations studied. The fluorescence intensity was quantified using the software *ImageJ*. The procedure was repeated after 1, 2, 3, 5 and 24 h using freshly prepared samples each time to avoid photo-bleaching of the Hoechst 33258 dye due to repeated UV irradiation.

In a different experiment, the MPE droplets were imaged after 24 h of gel formation to assess the mechanical resistance of their gelled core against the surfactant-mediated destabilization of the particle shell. To this aim, single MPE droplets (310 U mL^{-1} of alkaline phosphatase in carbonate buffer pH 10.2) after phagocytosis of the Fmoc-TyrP (20 mM) colloidosomes, were pipetted and deposited on a Petri dish containing 1 mL of dodecane. After that, 10 μL of a 15 mg mL^{-1} solution of oleic acid in dodecane were added to the proximity of the magnetic droplets and the behaviour of the sample was monitored using optical microscopy. The robustness and structural integrity provided by the Fmoc-TyrOH gel was also evaluated by pipetting the droplets after 24 h of gelation and placing them on an empty Petri dish to allow for overnight drying, followed by optical microscope imaging. Control experiments using non-gelled MPE droplets were also undertaken in both types of experiments.

Scanning electron microscopy (SEM) of MPE droplets after hydrogelation. Scanning electron microscopy of the MPE droplets after hydrogelation of the aqueous core by the alkaline-phosphatase

encapsulated colloidosomes was conducted after 1 week of formation using a JSM IT300 microscope. Several MPE droplets were pipetted from the dodecane phase and deposited on sticky carbon pads glued to a SEM aluminium stub for drying overnight. Before imaging, the samples were sputter-coated with a layer of silver of 15 nm. Elemental analysis of the samples was undertaken using energy dispersive X-ray spectroscopy (EDX).

2. Supplementary Movies

Movie S1. Optical microscopy video showing the formation of oxygen bubbles within the interior of a single MPE droplet containing a 5 % H_2O_2 solution (pH 10.2), after phagocytosis of silica colloidosomes containing catalase (30 mg mL^{-1}) (yellow objects) in a 2 mg mL^{-1} solution of oleic acid in dodecane. An external magnetic field is applied to displace the magnetic droplet towards the silica colloidosomes. Engulfment of the colloidosomes results in the catalase-mediated decomposition of H_2O_2 into water and oxygen bubbles, which causes the MPE droplet to float above the polystyrene substrate after 15 s. Movie is shown at real-time speed.

Movie S2. Optical microscopy video showing the formation and growth of oxygen bubbles within the interior of a buoyant MPE droplet (5 % H_2O_2) after phagocytosis of silica colloidosomes containing 30 mg mL^{-1} of catalase. Even though H_2O_2 decomposition takes place within the aqueous phase of the magnetic compartment, some bubbles escape through the oil/water interface, which could be attributed to the decrease of the interfacial tension by the oleic acid molecules. No magnetic field is applied. Movie is shown at real-time speed.

Movie S3. Optical microscopy video showing the buoyant, vertical motion of several 5% H_2O_2-containing MPE droplets after landing on a field of silica colloidosomes containing 30 mg mL^{-1} of a catalase solution (sediment at the bottom of the cuvette) in a dodecane-based oleic acid solution (2 mg mL^{-1}). Engulfment of the colloidosomes (as shown in Movie S1) results in oxygen bubble formation associated with H_2O_2 decomposition, and causes the MPE droplets to rapidly float away from the substrate. Vertical movement of the MPE droplets away from the colloidosomes arrests phagocytosis of the remaining population of the silica microstructures. Movie is shown at 25% of real-time speed.

Movie S4. Optical microscopy video showing the formation of particle-free apertures in the initially intact shell of a MPE droplet (0.1 M carbonate buffer pH 10.2) placed in a 10 mg mL^{-1} triolein solution, after the addition of multiple silica colloidosomes carrying a 100 U mL^{-1} lipase solution. The particle-free domains are associated with the release of surface active molecules such as mono-, diacylglycerols and free oleic acid from the hydrolysis of triolein by the lipase. The silica colloidosomes in contact with the particle-free patches of the magnetic droplet are spontaneously transferred to the aqueous phase, thus triggering their own phagocytosis. No magnetic field is applied. Movie is accelerated as indicated in the video.

Movie S5. Optical microscopy video showing the formation of oxygen bubbles within the interior of an initially intact single MPE droplet containing H_2O_2 (5 %, 1 M carbonate buffer pH 10.2) in a triolein solution (40 mg mL^{-1}) after the addition of silica colloidosomes containing both catalase (30 mg mL^{-1}), and lipase (200 mg mL^{-1}). Buoyancy of the MPE droplet is observed after *ca.* 40 s after the addition of the colloidosomes. The colloidosomes trigger their own phagocytosis, induce buoyancy, and remove the MPE host population from the substrate. No magnetic field is applied. Movie is shown at real-time speed.

3. Supplementary Figures

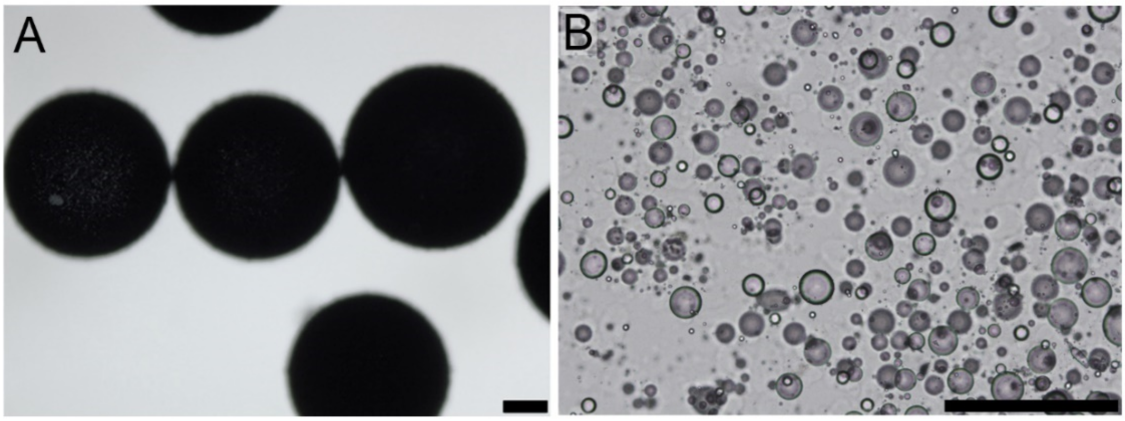

Fig. S1. Optical microscopy images of bioinorganic protocells in oil: magnetic Pickering emulsion (MPE) droplets **(a)** and silica colloidosomes **(b)**. The diameter of the MPE droplets (500 ± 250 μm) is *ca.* 10 times larger than that of the silica colloidosomes (52 ± 10 μm). Scale bars = 100 μm **(a)** 400 μm **(b)**. White balance correction has been applied to **(b)**.

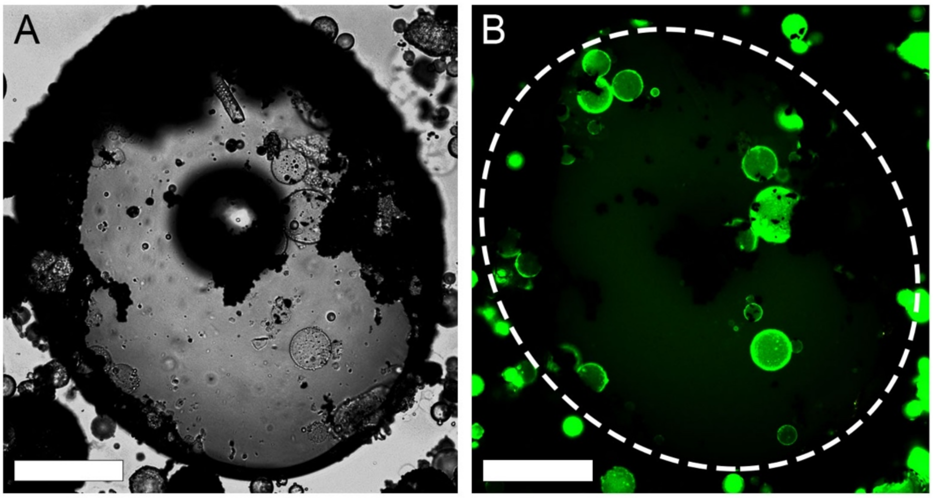

Fig. S2. Bright field **(a)** and fluorescence **(b)** microscopy images of a MPE droplet (delineated by a dashed line in (b)) after phagocytosis of several silica colloidosomes containing an aqueous solution of FITC-tagged catalase. The release of fluorescence from the engulfed silica colloidosomes is minimal, indicating that the enzyme mostly remained encapsulated within the interior of the phagocytosed silica colloidosomes. Scale bar = 200 μm.

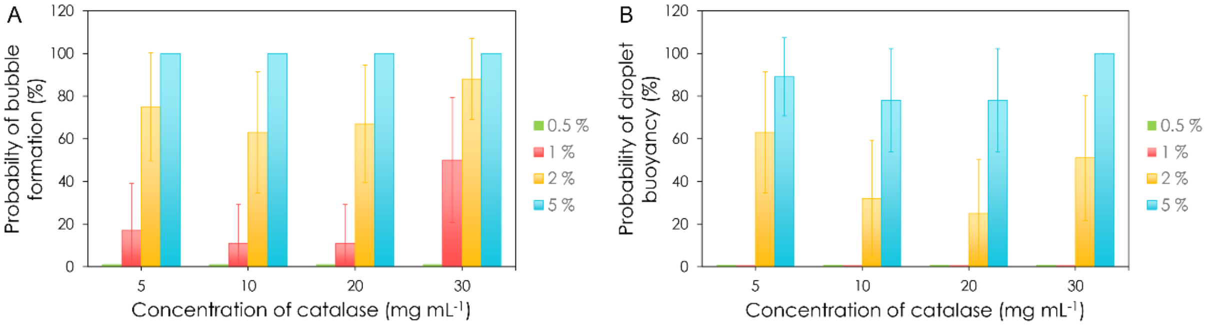

Fig. S3. Plots showing the probability of oxygen bubble formation inside MPE droplets containing solutions of H_2O_2 **(a)**, and the probability of MPE droplet buoyancy **(b)**, as a function of the concentration of the catalase solutions entrapped inside the silica colloidosomes. The different columns correspond to different concentrations of H_2O_2 (as indicated). Bubbles were only formed for concentrations of H_2O_2 larger than 1 % for all the assayed catalase concentrations. However, the level of bubble generation was insufficient to induce buoyancy of the MPE droplets for H_2O_2 concentrations lower than 2 %. The probabilities of bubble generation and buoyancy both increased with an increase in both the catalase and H_2O_2 concentrations. Error bars are based on binomial proportion confidence intervals.

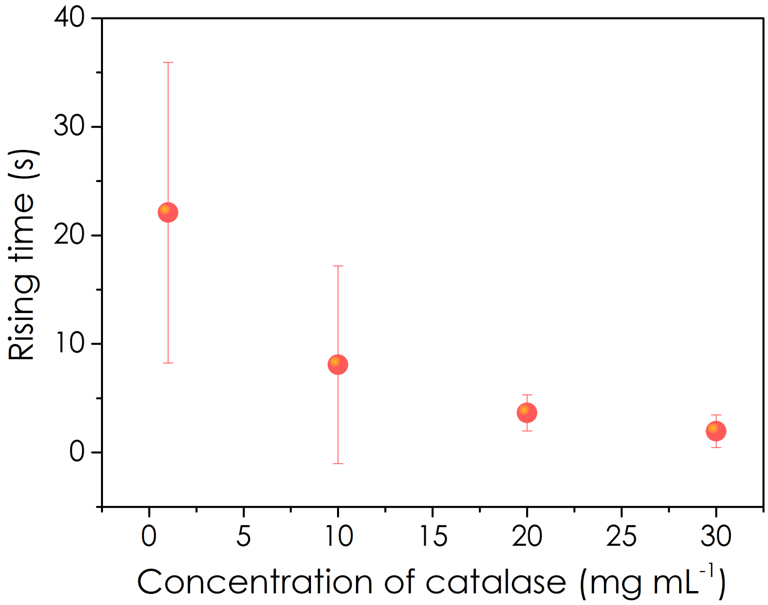

Fig. S4. Plot of the time elapsed for MPE droplets filled with 5 % H_2O_2 to rise above a field of catalase-containing colloidosomes (buoyancy) as a function of the catalase concentration. The droplets ascended faster for increasing concentrations of catalase, with rising times of less than 5 seconds for catalase amounts of 20-30 mg mL^{-1}. Error bars correspond to standard deviations.

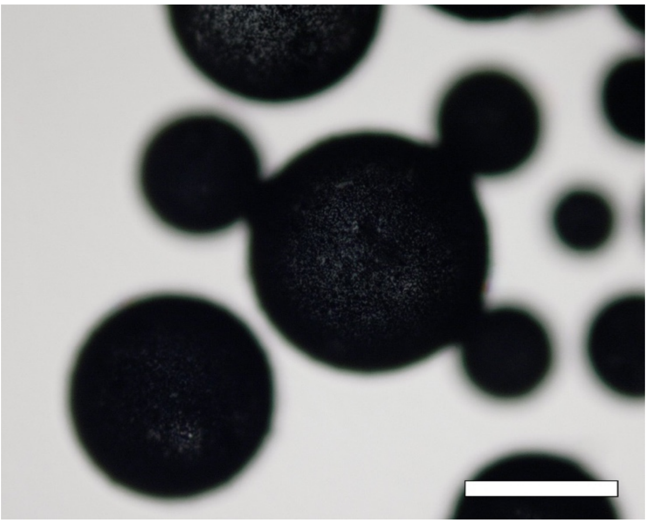

Fig. S5. Optical microscopy image of MPE droplets after 5 days of dispersion in a 20 mg mL^{-1} solution of triolein in dodecane. The droplets remained structurally stable with no changes in the magnetic shell. Scale bar = 250 μm.

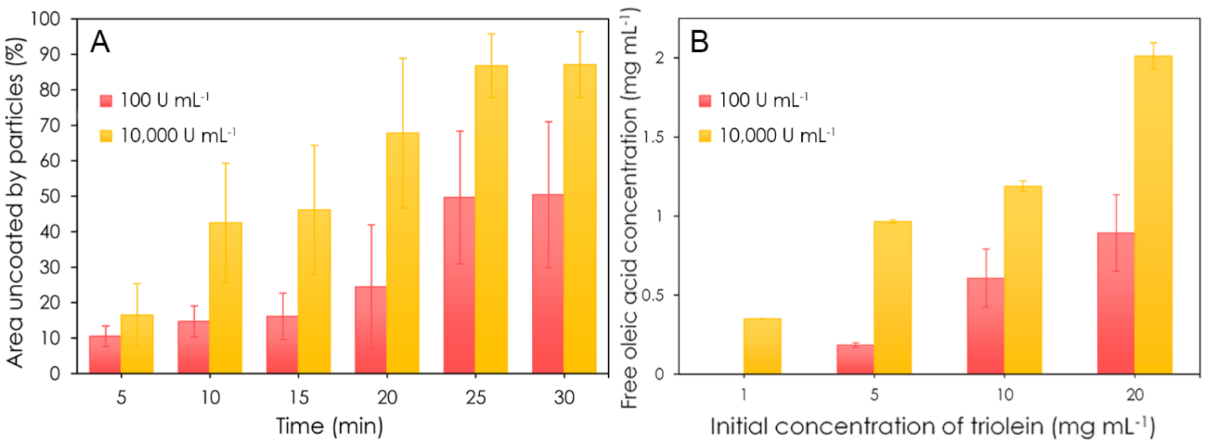

Fig. S6. (a) Plot showing changes over time in the percentage of surface area corresponding to the particle-free domains in the membrane of MPE droplets (carbonate buffer pH 10.2) initially dispersed in a 5 mg mL^{-1} solution of triolein in dodecane, after the addition of silica colloidosomes containing 100 (red columns) or 10,000 (yellow columns) U mL^{-1} of lipase solutions. Particle-free domains appeared as a consequence of the displacement of the magnetic particles by surface active molecules released by the lipase-mediated hydrolysis of triolein. The relative surface area of the particle-free patches increased over time, this effect being stronger the higher the concentration of lipase inside the silica colloidosomes. **(b)** Plot of the concentration of free oleic acid released into the dodecane medium after the hydrolysis of triolein by the lipase-containing colloidosomes (100 U mL^{-1}, red columns, or 10,000 U mL^{-1}, yellow columns) *vs* the initial concentration of triolein. Higher contents of the lipase resulted in higher oleic acid concentrations. Error bars correspond to standard deviations.

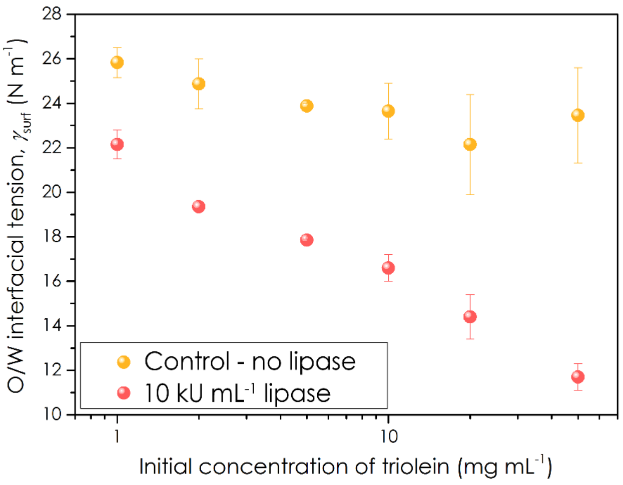

Fig. S7. Plot of the oil/water interfacial tension between an oil phase consisting of the supernatant of dispersions of lipase-containing colloidosomes after the hydrolysis of triolein, and carbonate buffer (pH 10.2), as a function of the initial concentration of triolein (*i.e.*, prior to colloidosome addition). The supernatant was diluted in dodecane with a 1:100 dilution ratio. The decrease of the interfacial tension with respect to control experiments in the absence of colloidosomes was caused by the release of surface-active molecules (mono-, diacylglycerols and free oleic acid) associated with the hydrolysis of triolein, catalysed by the lipase. The decrease of the interfacial tension was increased at high initial concentrations of triolein. Bars on data points represent standard deviations.

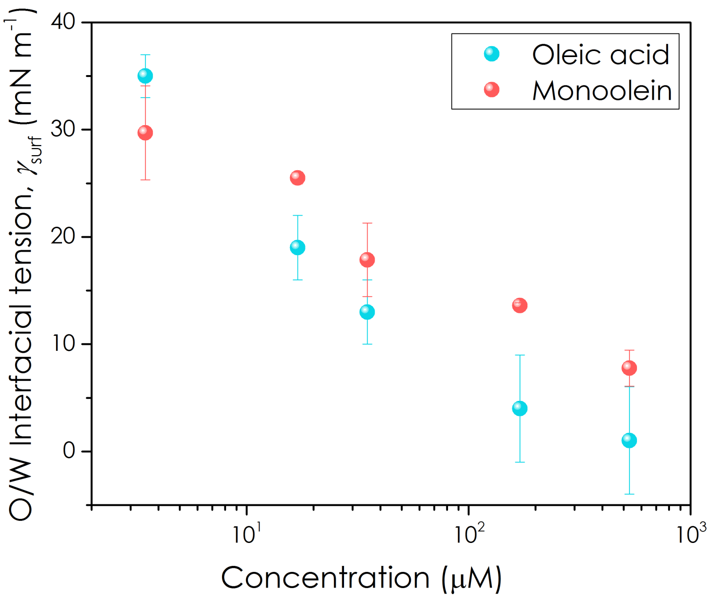

Fig. S8. Plot of the oil/water interfacial tension between carbonate buffer (0.1 M, pH 10.2) and solutions of oleic acid or monoolein in dodecane against the concentration of the surfactant in the oil phase. The value of the interfacial tension for pure dodecane was 40.2 ± 0.3 mN m^{-1}. The interfacial tension decreased with increasing surfactant concentration by a similar extent for both oleic acid and monoolein. Bars on data points represent standard deviations.

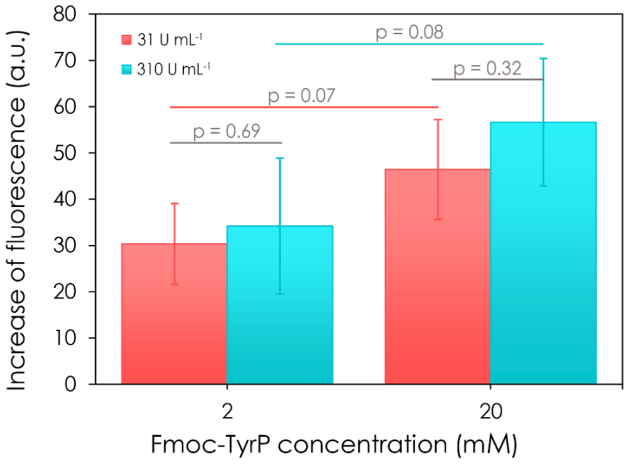

Figure S9. Plot showing the increase of blue fluorescence intensity (Hoechst 33258-hydrogel binding) inside MPE droplets against the concentration of encapsulated Fmoc-TyrP. Data were recorded after 24 h following the phagocytosis of silica colloidosomes containing ALP. Red and blue bars correspond to ALP concentrations of 31 and 310 U mL^{-1} respectively. Although the average fluorescence intensities increased with increasing enzyme and substrate concentrations, statistical differences between the values were not significant ($p > 0.1$) or marginally significant ($0.05 < p < 0.1$) respectively (two-tailed t test).

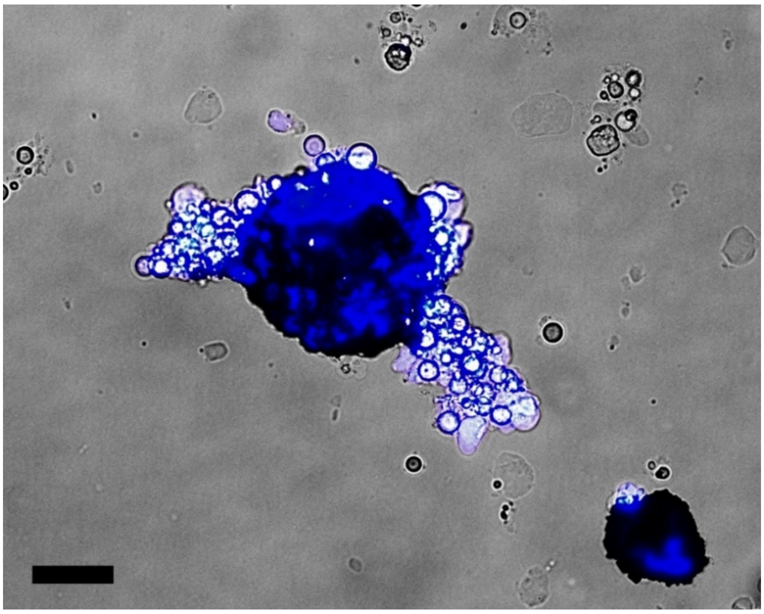

Fig. S10. Merged fluorescence/optical microscopy images showing two MPE droplets initially containing a Fmoc-TyrP solution of 10 mM and recorded 24 h after phagocytosis of ALP-entrapped silica colloidosomes (310 U mL^{-1}). The blue fluorescence is associated with binding of the dye Hoechst 33258 to Fmoc-TyrOH nanofilaments after ALP-mediated dephosphorylation. The larger MPE droplet is surrounded by aggregates of hydrogelated silica colloidosomes, suggesting that Fmoc-TyrP can access ALP in the partially-engulfed colloidosomes. White balance correction has been applied.

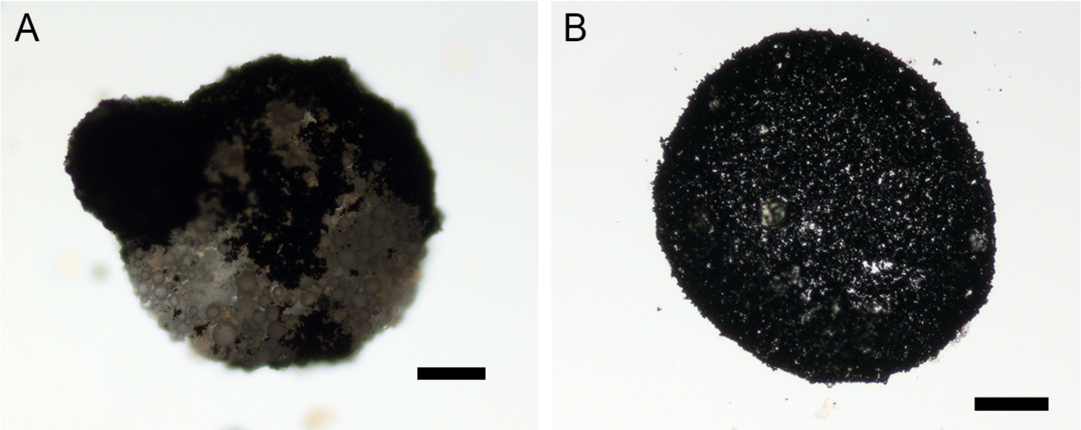

Figure S11. Optical microscopy images showing two dried single MPE droplets initially containing a Fmoc-TyrP solution: **(a)** after hydrogelation of the aqueous core caused by phagocytosis of multiple ALP-loaded silica colloidosomes, and **(b)** in the absence of phagocytosis. Unlike the native MPE droplet, the structure of the MPE droplet after hydrogelation is maintained and prevents the MPE droplet from collapsing upon drying. Scale bars = 100 μm. White balance correction has been applied.

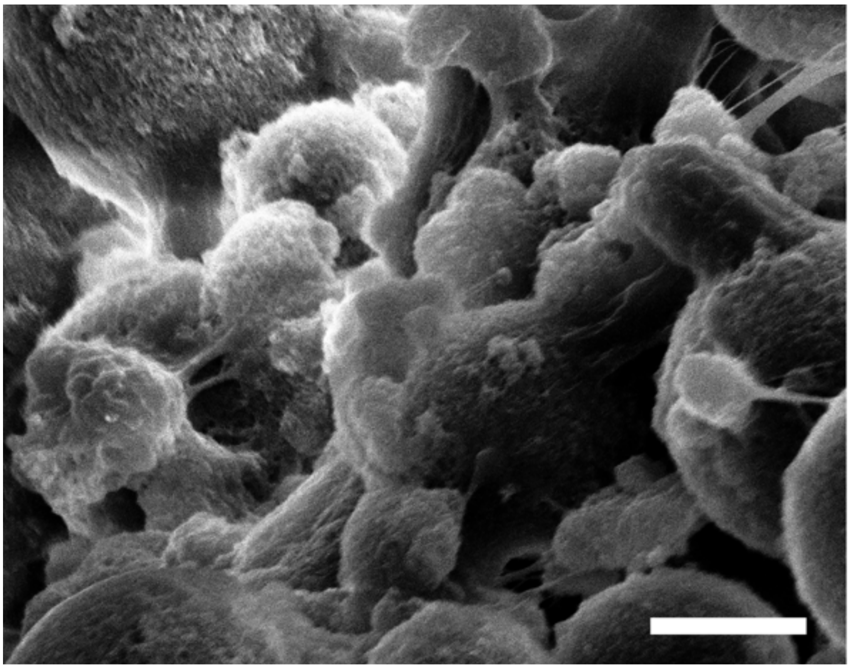

Figure S12. Scanning electron micrograph showing several phagocytosed ALP-encapsulating colloidosomes (31 U mL^{-1}) trapped inside a dried MPE droplet after 24 h. The silica colloidosomes are embedded in a filamentous hydrogel Fmoc-TyrOH network. Scale bar = 10 µm.
